# Profiling of antimicrobial dispensing practices in accredited drug dispensing outlets in Tanzania: a mixed-method cross-sectional study focusing on pediatric patients

**DOI:** 10.1186/s12913-022-08980-6

**Published:** 2022-12-23

**Authors:** David T. Myemba, Betty A. Maganda, Upendo O. Kibwana, Lilian Nkinda, Pacifique Ndayishimiye, Manase Kilonzi, Wigilya P. Mikomangwa, Belinda J. Njiro, Harrieth P. Ndumwa, Hamu J. Mlyuka, Fatuma F. Felix, Dorkasi L. Mwakawanga, Peter P. Kunambi, Godfrey Sambayi, Judith K. Costantine, Alphonce I. Marealle, Ritah Mutagonda, Gerald J. Makuka, Samson W. Kubigwa, Nathanael Sirili, Rogers Mwakalukwa, Rashid Mfaume, Arapha Bashir Nshau, George M. Bwire, Elevanie Nyankesha, Robert W. Scherpbier

**Affiliations:** 1 School of Pharmacy, Muhimbili University of Health and Allied Sciences, P.O. Box 65001, Dar Es Salaam, Tanzania; 2 School of Medicine, Muhimbili University of Health and Allied Sciences, P.O. Box 65001, Dar Es Salaam, Tanzania; 3 School of Medicine and Pharmacy, College of Medicine and Health Sciences, University of Rwanda, P.O. Box 4285, Kigali, Rwanda; 4 School of Nursing, Muhimbili University of Health and Allied Sciences, P.O. Box 65001, Dar Es Salaam, Tanzania; 5 Liwale District Hospital, P.O Box 28, Lindi, Tanzania; 6 School of Public Health and Social Sciences, Muhimbili University of Health and Allied Sciences, P.O. Box 65001, Dar Es Salaam, Tanzania; 7 Regional Administrative Secretary, P.O. Box 5429, Dar Es Salaam, Tanzania; 8 The Pharmacy Council of Tanzania, P.O. Box 31818, Dar Es Salaam, Tanzania; 9 United Nations Children’s Fund, 3 United Nations Plaza, New York, NY 10017 USA; 10 United Nations Children’s Fund, Bâtiment BIT, Route Des Morillons 4, CH-1211 Geneva, Switzerland

**Keywords:** Dispensing profiles, ADDOs, Dispensing practices, Antimicrobials, Children

## Abstract

**Background:**

The emergency of antimicrobial resistance due to irrational antimicrobial use has put public health under threat. Accredited Drug Dispensing Outlets (ADDOs) play an important role in enhancing availability and accessibility of antimicrobials, however, there is a scarcity of studies assessing antimicrobial dispensing practices in these outlets, focusing on children in Tanzania.

**Objective:**

This study was conducted to assess the antimicrobial dispensing practices among ADDO dispensers and explore the factors influencing the use of antimicrobials for children in Tanzania.

**Methods:**

A community-based cross-sectional study utilizing both qualitative (interviews) and quantitative (simulated clients) methods was conducted between June and September 2020 in seven zones and 14 regions in Tanzania.

**Results:**

The study found inappropriate dispensing and use of antimicrobials for children, influenced by multiple factors such as patient’s and dispenser’s knowledge and attitude, financial constraints, and product-related factors. Only 8% (62/773) of dispensers asked for prescriptions, while the majority (90%) were willing to dispense without prescriptions. Most dispensers, 83% (426/513), supplied incomplete doses of antimicrobials and only 60.5% (345/570) of the dispensers gave proper instructions for antimicrobial use to clients. Over 75% of ADDO dispensers displayed poor practice in taking patient history.

**Conclusion:**

ADDO dispensers demonstrated poor practices in dispensing and promoting rational antimicrobial use for children. Training, support, and regulatory interventions are required to improve antimicrobial dispensing practices in community drug outlets.

## Background

Resistance to antimicrobials is rampant and has emerged as a public health threat of the modern era. The 2019 World Bank review work on ‘pulling together to beat superbugs’ showed that 700,000 lives are lost yearly worldwide due to antimicrobial resistance [1] and the number is still on the rise.

Children are vulnerable to infectious diseases because of their immature immune system among other factors. In the year 2018, approximately 29 percent of global deaths among children under the age of 5 was caused by pneumonia, diarrhea, and malaria, of which the greatest percent was from sub-Saharan Africa [2]. Until recently, most antimicrobials were effective to eliminate the majority of the under 5 infections. However, the emergency of antimicrobial resistance due to irrational antimicrobial use and evolution of micro-organisms has rendered most of these previously effective drugs ineffective [3].

Although antimicrobials are classified as prescription-only medicines (POM), dispensing malpractice is a real challenge in most African settings [3]. Experience in Tanzania shows that most medicines, including antibiotics, can be bought from retail drug outlets without prescription [3]. This is expected to lead to overuse or inappropriate use of antibiotics, which in turn may lead to antimicrobial resistance (AMR). Some of the most common malpractices reported in Tanzania include; illegal stocking of prescription-only medicines, use of unqualified staff and referral by healthcare facility staff to private outlets in which they have financial stakes [3].

Mitigations to reduce the burden of disease, antimicrobial resistance, and mortality in sub-Saharan Africa (SSA) have increasingly recognized the important role of drug retailers in delivering basic healthcare services. Drug outlets comprise a fair portion (nearly 40%) of the private healthcare sector in SSA and provide between 15 and 83% of all child health services in some countries [4]. To address the role of the retail drug sector, several countries in SSA have included private drug retailers in national health interventions [4, 5].

In 2003, Tanzania launched an Accredited Drug Dispensing Outlet (ADDO) program, which combined owner and dispenser training. Accreditation of ADDO by regulatory bodies is based on standards, business incentives, and local regulatory enforcement, with efforts to increase consumer access to quality products and services. The primary goal of the program was to improve access to good-quality medicines in rural and peri-urban areas where there was frequent drug shortage in public healthcare facilities and few or no registered pharmacies [6, 7]. To date, ADDOs are the major source of medicines and primary health services for Tanzanians living in rural and peri-urban areas [5, 7]. According to a 2018 report by the Pharmacy council (PC) of Tanzania, there are about 11,356 ADDOs compared to 1334 registered retail and wholesale pharmacies [8]. Furthermore, there are about 19,000 trained ADDO dispensers in Tanzania [5, 9]. ADDO dispensers undergo short training mainly on dispensing practice, pharmacology, communication skills and, in addition, integrated management of childhood illnesses (IMCI), which includes principles of effective acute respiratory tract infection (ARI) and diarrhea management in children [9]. Earlier research has reported that ADDOs have increased accessibility to antimicrobials, and they have decreased the proportion of unauthorized medicines from 53 to 13% [9]. Given the fact that ADDOs are an important part of a multi-faceted health care system, while accreditation has attempted to address the quality of pharmaceutical services in private drug outlets, efforts to improve access to and use of medicines in Tanzania need to target ADDOs especially with focus on vulnerable groups such as children [10]. Considering their number, market share, and coverage in rural and peri-urban communities, ADDOs present an important opportunity for implementing antimicrobial stewardship programs (ASP) to curb the AMR nightmare. While several studies have been conducted to evaluate the influence of ADDO dispensers’ knowledge and practice on irrational antimicrobial use in adults [9, 11, 12], however, less has been done for the pediatric population. Chalker et al. reported an overuse of antimicrobials for children, contributed by poor prescribing and dispensing practices as well as inappropriate customer demand [13]. This study was conducted to assess the antimicrobial dispensing practices among ADDO dispensers and explore the factors influencing the use of antimicrobials for children in Tanzania. Furthermore, the study provides recommendations for strengthening the antimicrobial stewardship program in the country.

## Methods

### Study design, study sites and study population

**Fig. 1:**
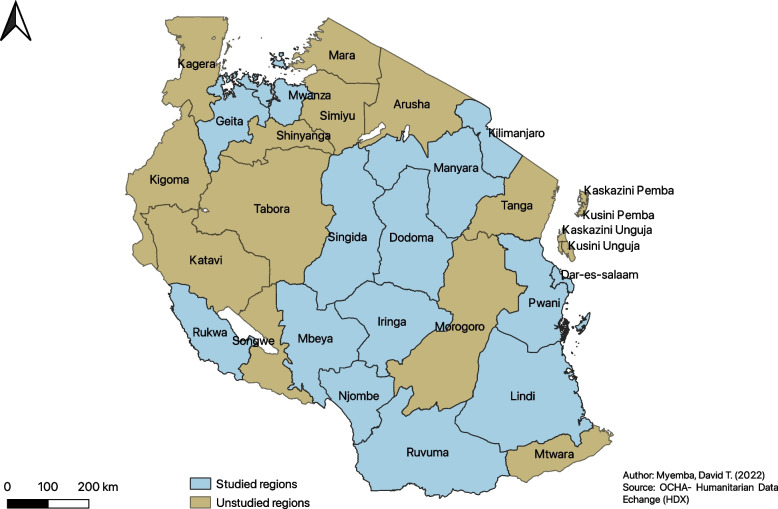
Tanzania’s Map indicating study sites (regions); Southern west highlands zone- Mbeya and Rukwa; Southern highlands zone- Iringa and Njombe; Lake zone- Geita and Mwanza; Central zone- Singida and Dodoma; Eastern zone- Pwani and Dar es Salaam; Northern zone- Manyara and Kilimanjaro; Southern zone- Lindi and Ruvuma

This was a community-based, cross-sectional study utilizing both quantitative and qualitative methods. Interviews were used to explore ADDO dispensers’ practices through focused group discussions (FGDs). Quantitative data were collected using simulated clients to assess the antimicrobial dispensing practices among ADDO dispensers. Method selection was inspired by similar studies done elsewhere [14, 15]. The study was conducted in fourteen regions from seven administrative zones in Tanzania mainland between June and September 2020. The regions were purposefully selected to get a representation of the diverse Tanzanian population from each zone (Fig. 1). Selection was based on longer time of the ADDO program implementation, high reported prevalence of irrational antimicrobial use [12] and lower multidimensional poverty index [16] for regions like Mbeya, Njombe, Iringa, Ruvuma, Tanga, Kilimanjaro and Mwanza. Dodoma and Singida represented regions with higher multidimensional poverty index [16]. Dar es salaam was selected based on a reported high antimicrobial resistance patterns and high levels of irrational antimicrobial use [17]. The target population were ADDOs and ADDO dispensers in the regions selected to represent each zone. The number of ADDOs studied in each region was determined by the total number of registered ADDOs available in the region.

### Data collection procedures

#### Quantitative study

A total of 805 simulated client visits were conducted in this study to assess the antimicrobial dispensing practices among ADDO dispensers. The sampling technique used was adopted from a previous study on the assessment of the application of basic pharmacology and dispensing practices of ADDO dispensers [3] where a total of 104 ADDO dispensers were recruited to represent the study population. For this study, we customized the sample size of 104 to represent the minimum number of dispensers in each zone (104*7), therefore we had a minimum total of 728 simulated client visits. The actual number of visits in each of the fourteen regions depended on the proportion of registered ADDOs in the region relative to the national number. The simulated client visits were divided among three assessment scenarios: sneezing and runny nose (an upper respiratory tract infection, URTI), childhood non-bloody diarrhea and a request for co-trimoxazole. The first two scenarios were chosen because they are the symptoms most associated with misuse of antibiotics [18]. Co-trimoxazole (commonly known locally by a brand name Septrin®) is a prescription only medicine (POM) and one of the most used antimicrobials for both adults and children (for UTI) locally. A request for this antibiotic was unlikely to raise suspicion by the dispensers. Simulated clients posed as parents/guardians of a 5-year-old child with sneezing or diarrhea and then asked for medicines after a conversation with the dispensers. Similarly, the simulated clients requested co-trimoxazole (without a prescription) for a 5-year-old child. During a visit, a simulated client assessed whether the dispenser; asked about duration of child’s illness, history of similar illness, if the child had used other medicines before for similar illness, if the client had visited a health care facility for the similar illness previously, the child’s age, for a prescription, was willing to dispense antimicrobials without a prescription, was ready to dispense incomplete dose, and/or give proper instructions on how to use the antimicrobials upon dispensing. These assessment criteria constituted a total of nine items on a simulated client checklist. The first seven items were designed to assess the dispenser’s practice in taking patient’s medical and medication history and requesting for prescription while the last two were designed to assess the dispenser’s practice in dosing and patient counseling. After each visit, the simulated clients immediately completed a data collection tool (checklist) away from the site. The forms were then sent online via an Open Data Kit (ODK Software, USA). To avoid suspicion, simulated clients did not re-visit an ADDO with a similar or different scenario.

#### Qualitative study

At least one focused group discussion (FGD) was conducted in each of the selected regions, 6–10 ADDO dispensers were recruited for the FGDs in each region to explore their practice on rational antimicrobial use. Sampling was purposeful and we used the principle of saturation to ensure that we obtain adequate data for the respective questions in the interview guides. The interview guide was developed following extensive literature review and expert consultation. The guide consisted of two parts; part one consisted of participant’s sociodemographic information and part two consisted of a total of ten guiding questions. The questions were focused on exploring situations prompting the dispensing of antimicrobials, the dispensing practices and efforts taken to promote rational antimicrobial use.

#### Training and pre-testing of data collection tools

Investigators conducted an initial training of a team of data collectors on proper scenario presentation and probing of interview questions. Prior to data collection, a pilot study was conducted to validate the data collection tools and procedures and to train the data collection team, which consisted of about 17 researchers, 4 of which were based in Dar es Salaam, and one in each of the rest of the regions. This team consisted of researchers with pharmaceutical/medical background. During the pilot study, the researchers visited several selected ADDOs to simulate the scenarios and took part in focused group discussions (FGDs) with selected ADDO dispensers. The simulated visits took about half a day per day, while the FGDs took between 30 and 60 min. Necessary modifications and changes were made based on feedback from these pilot visits and interviews. All the ADDOs involved in the pilot study were excluded in the actual study.

### Data management and analysis

#### Quantitative data analysis

The simulated client (checklist) data were exported to Microsoft Excel Sheet (Redmond, WA) then to statistical package for social sciences version 25 (SPSS Software, Chicago Inc., USA) for coding and analysis. The data were presented using descriptive statistics (frequencies and percentages). Furthermore, in the simulated client data, a dispenser’s right practice for each of the assessment criteria was given a score of one ‘1’ while a wrong practice was given a score of zero ‘0’. The practice scores were summarized using median and interquartile range (IQR). Furthermore, scores from the nine criteria were summed up to make the final score for each dispenser (maximum score = 9). The overall dispensing practice was categorized as poor practice (when the final score was less than 6) and good practice (when the final score was equal to 6 and above).

#### Qualitative data analysis

**Fig. 2:**
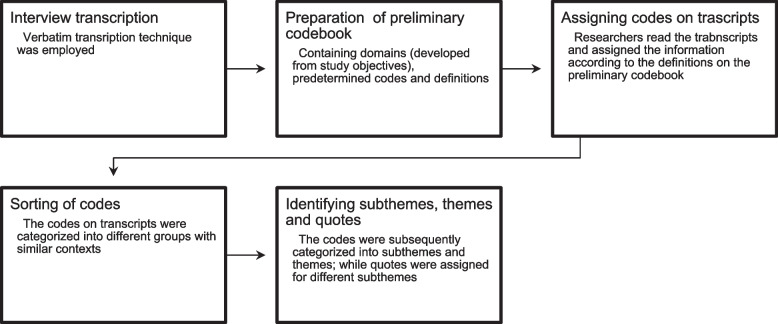
Qualitative data analysis process

A thematic analysis approach was used to analyze the information following the five stages as described by Braun and Clarke, 2014 to establish meaningful patterns in the data: familiarization with the data, generating initial codes, searching for themes among codes, reviewing themes and presenting the results [19]. Results were organized into themes, subthemes, and quotes. Please refer to Fig. 2 for a detailed qualitative data analysis process.

## Results

### Quantitative study findings

#### Scenario-based practice of ADDO dispensers in individual dispensing criteria

About 280 (34.8%) dispensers were reached for the sneezing and runny nose scenario. Majority of the dispensers, 94.6% (*n* = 265), asked about the child’s age while just 12.1% (*n* = 34) asked whether the client had visited a health facility for the same illness. Only 6.8% (*n* = 18) asked for a prescription and 82.9% (*n* = 209) were willing to dispense without prescription.

About 265 dispensers (32.9%) were reached for the non-bloody diarrhea scenario. About 95.8% (*n* = 254) of the dispensers asked for the child’s age while fewest, 13.0% (*n* = 34), asked whether the child had a history of similar illness and 18.6% (*n* = 49) asked for a previous visit to a health facility for the same illness. Only 11.1% (*n* = 29) asked for a prescription and 93.9% (*n* = 246) were willing to dispense without prescription.

About 260 dispensers (32.3%) were reached for the co-trimoxazole request scenario. Only 6.0% of the dispensers (*n* = 15) asked for a prescription and most of the dispensers 91.8% (*n* = 235) were willing to dispense without prescription.

Almost equal proportions of dispensers in the sneezing 60.1% (*n* = 98), diarrhea 62.6% (*n* = 132) and Co-trimoxazole request 58.7% (*n* = 115) scenarios gave proper instructions upon dispensing antimicrobials to the clients (Table 1).

**Table 1:** Dispensing practice of ADDO dispensers based on simulated scenarios

Dispenser asked about (Yes response)	Sneezing and runny nose n (%)	Non-bloody diarrhea n (%)	Co-trimoxazole request n (%)
Duration of illness	146 (52.1)	188 (71.5)	53 (21.9)
Previous medication for same illness	67 (24.0)	64 (24.2)	17 (7.1)
History of similar illness	41 (14.6)	34 (13.0)	13 (5.5)
Previous visit to health facility for the similar illness	34 (12.1)	49 (18.6)	17 (7.1)
Prescription	18 (6.8)	29 (11.1)	15 (6.0)
Child’s age	265 (94.6)	254 (95.8)	161 (66.3)
Dispenser was willing to dispense without prescription	209 (82.9)	246 (93.9)	235 (91.8)
Dispenser was ready to dispense incomplete dose	102 (76.1)	145 (82.9)	179 (87.7)
Dispenser gave instruction on how to use the antimicrobials?	98 (60.1)	132 (62.6)	115 (58.7)

#### Overall antimicrobial dispensing practices of ADDO dispensers based on the simulated scenarios

A total of 805 ADDO dispensers were reached in the simulated client study. In general, out of 773 dispensers who were able to dispense medicines, only 8.0% (*n* = 62) asked for a prescription, 83.0% of 513 (*n* = 426) were ready to dispense incomplete doses of antimicrobials, while only 60.5% of 570 (*n* = 345) gave proper instructions on how to use the antimicrobials upon dispensing (Table 2).

**Table 2:** Overall summary of the practice of ADDO dispensers in individual assessment criteria

Assessment criterion	Frequency (n) ^1^	Percentage (%)	*P*-value
Dispenser asked about duration of illness	386	49.5	0.92
Dispenser asked about previous medicines for the similar illness	148	19.0	< 0.001
Dispenser asked about history of similar illness	88	11.3	< 0.001
Dispenser asked about previous visit to health facility for the similar illness	100	12.8	< 0.001
Dispenser asked about child’s age	680	86.3	< 0.001
Dispenser asked about asked for prescription	62	8.0	< 0.001
Dispenser was willing to dispense without prescription	690	89.6	< 0.001
Dispenser was ready to dispense incomplete dose	426	83.0	< 0.001
Dispenser gave proper instructions on how to use the dispensed antimicrobials	345	60.5	0.04

^1^Frequency for the ‘yes’ response or right practice

**Fig. 3:**
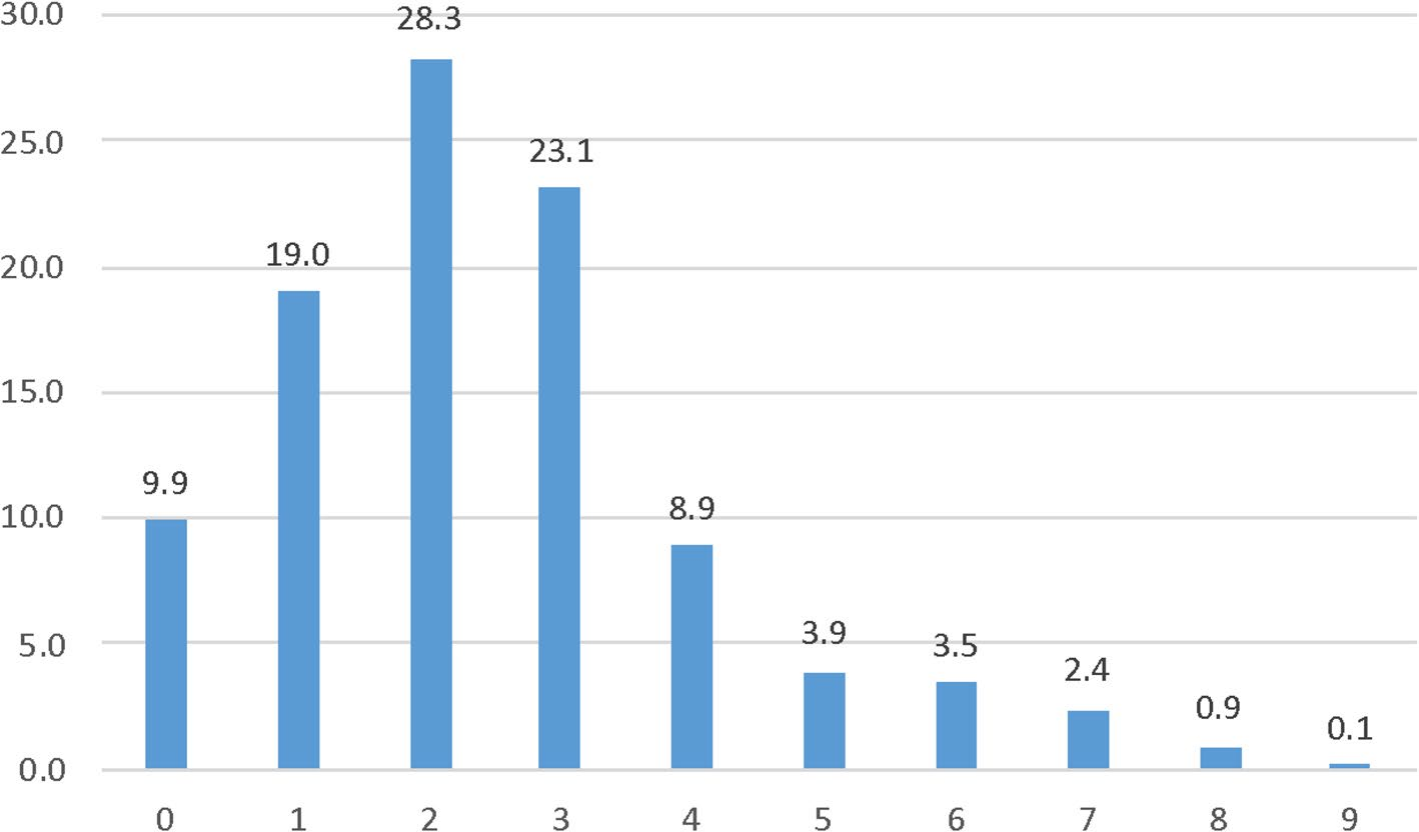
Proportional (percent) distribution of practice scores among the ADDO dispensers. Note that the distribution is screwed to the left

In general, the ADDO dispensers displayed poor dispensing practices, with the practice scores distribution highly skewed to the left (Fig. 3). The median practice score was 2.0 (IQR: 1.0 – 3.0). Majority of the dispensers, 93.2% (750/805) had poor practice.

#### Qualitative study findings

The following themes emerged from the discussions with the ADDO dispensers on the dispensing practices and use of antimicrobials.

#### Multiple factors influencing the dispensing of antimicrobials

##### Patients’ preference for certain types of antimicrobials

Participants of this study stated that they often dispense antimicrobials based on patient preferences. Participants explained that patients have names for the antimicrobials that they want when they visit ADDOs, based on recommendations from relatives or friends. Furthermore, participants added that patients believe that certain antimicrobials can treat all signs and symptoms, and this makes them reluctant to accept advice from healthcare providers. One dispenser stated;

“… Most clients select medications based on experience. Example, currently amoxicillin and ampicillin are not preferred regardless of whether the dose is sold at a high or low price, for now the preferred medication is ampiclox, every patient who comes would refuse a recommendation of ampicillin, saying they want the drug with purple and black color (ampiclox). That gives us a hard time educating them”. (FGD member no.4- Manyara).

Another dispenser added;

“As previously stated, let’s say it is what the buyer of the medication trusts. When someone comes from their house knowing they are going to buy a certain medication, changing their mind is so difficult”. (FGD member no.4- Songwe).

##### Financial constraints facing the patients

The dispensers admitted that they were forced to dispense certain antimicrobials based on the financial status of the patient. They stated that patients could not afford antimicrobials that are expensive for them and demanded a cheaper alternative. One participant said;

“…… You find a person waking up in the morning with two thousand TZS (< 1 USD) only, children have not eaten, and the symptoms have relieved after using only five tablets out of the required full dosage. Even if they wish to finish the dose, they will not be able to, because the children must eat as well”. (FGD member no.8- Kibaha).

Another participant said;

“It is true they come to the shop and you explain to them that they must finish the dose. Some would say I only have half the money, so give the half dose first and then I will take the remaining half later. But when you give them the half dose, they do not return to the shop when their symptoms disappear”. (FGD member no.6- Rukwa).

##### ADDO owners influence dispensing practices

Participants explained that dispensing of antimicrobials in ADDO was influenced by business perspectives of the facility leaders/owners. They stated that sometimes ADDO owners forced the dispensers to dispense medicines inappropriately to maximize profit, as one dispenser revealed;

“Owners of ADDO shops are the ones who can contribute to distorting service provision, because they believe in selling incomplete doses and I as an employee do not have a voice because it is not my shop, the result could be that I lose my job and another dispenser will be employed”. (FGD member no.5- Geita).

#### Factors influencing adherence to antimicrobial treatment among patients

##### Instructions on antimicrobial use to patients by health care workers

Participants stated that instructions from prescribers or dispensers on how to use antimicrobials had great influence on the use of antimicrobials by the patients. They added that adequate and appropriate instructions on adverse/side effects and warnings on antimicrobial use, influence the use of antimicrobials and thus have an impact on antimicrobial resistance. One dispenser stated;

“Even if the dispensed medication is for the child, I will insist the mother on the necessity of completing the dose. And if I want to make further follow up, I will ask the mother to return after several days to check if the dose was really finished. If there is any other problem, I will advise them again”. (FGD member no.4- Dodoma).

Another dispenser added;

“For me, I usually advise my clients that if you take this medication and you feel it does not suit you, return so that I can change it for you, because sometimes certain medications contain Sulphur, and they might harm the patient. Or others develop rashes…. Others vomit, that is why I tell them that if you get any side effects, return so that I change the mediation”. (FGD member no.2- Iringa).

##### Product factors (dose and dosage)

Participants explained that product characteristics such as size of the tablet, number of tablets per dose, smell and taste of the tablets and nature of the side effects influenced patients’ selection and use of antimicrobials. One of the participants said;

“In addition, depending on the taste of the medication, some fail to complete the dose… some medications have a bitter taste, others have an irritating smell… the other reason is the size of the tablet that the person should take and the number of the tablets that they are given”. (FGD member no.5-Kibaha).

Another participant exemplified the influence of adverse effects;

“Then there is Septrin also, most people do not use this antibiotic because they are allergic to it… some either experience itching or peeling of the skin, so it is a drug that is hardly being sold”. (FGD member no.1- Kilimanjaro).

##### Work/school schedule as a hindrance to treatment adherence

Participants explained that parents faced challenges to administer antimicrobials to their children during work/school hours. They added that most of the parents demanded antimicrobials which could be taken once or twice a day, for them to be able to supervise and monitor uses of the medications, as revealed by one of the participants;

“Some say because they go to work and leave the child with maybe the house maid or someone else, so if the medication dose requires taking it during the afternoon hours, most of these doses are usually skipped unless the mother makes a phone call”. (FGD member no.3- Dar es salaam).

##### Poor knowledge on the importance of dose completion

Participants stated that poor knowledge among patients on the importance of dose completion and consequences of not taking the whole dose contributed to the behavior of patients not completing medication courses. In addition, due to poor knowledge, the majority of their patients stopped using medications when signs and symptoms were resolved, and some could not follow the instructions given by health care providers. One dispenser revealed;

“A patient may come to you requesting for two tablets just to bring relief to what is disturbing them… instead, you advise and dispense to them the complete dose, and still they will complain that they cannot complete the dose saying it’s a lot, and since most doses for antibiotics is 30 capsules, they would ask, “where in my body will I put all 30 capsules? I cannot finish this medication”. (FGD member no.4-Kilimanjaro).

## Discussion

The study found that ADDO dispensers generally had poor antimicrobial dispensing practices. In all the simulated scenarios, more than 90% of all the dispensers displayed poor practice in taking patient’s medical and medication history and requesting prescriptions. Majority of the dispensers did not ask about duration of child’s illness, history of similar illness, whether the child had used other medicines before for similar illness and/or if the client had previously visited a health facility for similar illness. Majority of them, however, asked about the child’s age. Majority did not ask for, and were willing to dispense antimicrobials without a prescription.

These findings are consistent with other studies. A systematic report of global access to antibiotics found a pooled proportion of non-prescription supply of antibiotics as large as 62% globally [20]. Another systematic review of 50 studies reporting non-prescription sales of antimicrobial agents at community pharmacies in developing countries found non-prescription supply of antimicrobials was reported in 28 developing countries across Asia, Africa, South America, Europe, and Middle eastern regions [21]. Similarly, antimicrobials were obtained without prescriptions and/or for conditions for which no antibiotics are generally needed such as upper respiratory tract infection symptoms and acute childhood diarrhea in several other studies [13, 22–26]. Incomplete taking of patient history was reported in other studies from low- and middle-income Asian settings [27]. In another study in Tanzania, most dispensers did not ask any of the shoppers about danger signs potentially associated with pneumonia in a child [26]. Similarly, in an Ethiopian study, dispensers did not ask about past medical and medication history and patient’s history of drug allergy [23]. Poor practice in taking patient’s medical and medication history has been reported in other studies as well [13, 24–28].

In this study, majority of the dispensers dispensed incomplete doses of antimicrobials. Similar practices were reported in Tanzania and other places before. A recent study among community drug retailers in Moshi Tanzania reported that prescriptions with incomplete doses were accepted and had antibiotics dispensed [29]. Elsewhere, most community pharmacies in central Nepal dispensed incomplete doses of antibiotics [30]. Dispensing incomplete doses of antimicrobials has been reported in other studies in Tanzania before [3, 31]. Furthermore, the study found that the majority of the dispensers had poor counselling practices as they did not provide proper drug information to their clients when dispensing antimicrobials. Similarly, a study conducted in Moshi, Tanzania found only 5.9% of drug retailers gave instructions for medicine use, while none of the retailers explained drug side-effects [29]. There have been reports of similar practices in Africa and other parts of the world [24, 28, 30].

The poor dispensing practices reported in this study constitute an irrational use of antimicrobials, a main contributing factor to antimicrobials resistance [32–34]. Tanzania's National Action Plan on Antimicrobial Resistance (NAPAR) identifies irrational use of antimicrobials as one of the areas to be addressed to tackle this wicked health problem [35].

Findings from the qualitative study show that the poor antimicrobial dispensing practices were influenced by financial constraints facing the patients, which prompted dispensing of incomplete doses; patients’ preferences for certain antimicrobials; motivation to generate income; a communication gap among health care workers and non-adherence to dispensing guidelines. Similarly, factors influencing improper antimicrobial dispensing practices among community drug retailers have been reported in other studies. A qualitative study among accredited drug dispensing outlets in Tanzania revealed dispensers’ behavior was driven by customer demand, habit (“mazoea”), following inappropriate health facility prescriptions, and the need to make a profit [11]. Another report cited poor national medicine regulations, limited availability of qualified pharmacists, commercial pressure on pharmacy staff, consumer demand, inappropriate prescribing practices, and a lack of awareness of AMR as contributing factors [21]. Similarly, patient request and community pharmacy staff recommendation have been reported as influencing factors to non-prescription supply of antibiotics [20]. Other factors reported to influence antimicrobial dispensing practices include but are not limited to having non-pharmacist staff [30], perceived financial benefit, high expectation and/or demand of customers and competition among pharmacies [23], prescriptions presented by clients, patients’ finances, and patient preferences [36], lack of controls in the dispensing of antibiotics and poverty [22], lack of knowledge among dispensers and poor regulatory environment [27] and false feeling of being qualified, social acceptance, customer demands, public beliefs, high consultation fees of doctors, expensive diagnostic tests and economic influences [37].

The lack of knowledge of antimicrobials and/or AMR among dispensers as a contributing factor to inappropriate dispensing of antimicrobials is further vindicated by the influence that training may have on the dispensers’ practice. A study among ADDO dispensers and community members in Kilosa district in Tanzania showed the percentage of ADDO dispensers following good dispensing practices increased from the first monitoring visit to the last visit after providing training and on-site support [16]. In the same intervention, more dispensers could name more factors contributing to AMR and negative consequences of inappropriate antimicrobial use, and most ADDO customers knew important information about the medicines they were dispensed [17].

The reported poor antimicrobial dispensing practices of ADDO dispensers and the factors influencing their dispensing practices continue to emphasize that combating AMR needs a transdisciplinary approach, centered on enhancing education and providing training and support to both service providers and the community.

The take-home message from this study is very clear. There is an inappropriate dispensing and use of antimicrobials for children in our settings. These inappropriate dispensing and use practices are influenced by multiple factors such as patient’s and dispenser’s knowledge and attitude, financial constraints, and product-related factors. However, these results should be interpreted with caution. First, it was not statistically possible to determine the factors associated with the poor dispensing practices observed in the quantitative study, since the use of simulated clients limited the possibility of gathering social demographic information of the ADDO dispensers. Such information as age, professional education level and experience at work, would have made it possible to draw some useful inferential statistics. Second, although the qualitative findings provide a useful insight into the factors influencing the dispensing practices (that would otherwise be difficult or even impossible to explore with a quantitative approach), these findings are context- and perspective- based and lack generalizability. Third, it was difficult to interpret the quantitative and qualitative findings in conjunction as different sets of dispensers were involved in the two studies. The simulated client approach made it difficult to involve the same dispensers in the two studies, yet it was necessary to capture the real dispensing practice. Future studies should maximize the potential for deep insights from qualitative methods while also using quantitative methods to obtain more generalizable results. This means that larger, mixed-method studies, possibly involving a similar set of dispensers, are highly recommended.

## Conclusions

Majority of the ADDO dispensers had poor antimicrobial dispensing practices, which included not taking patient’s history, dispensing without prescriptions, dispensing incomplete doses and not doing proper patient counselling. Poor dispensing practices were influenced by such factors as patient’s preference on certain types of antimicrobials, financial constraints facing the patients, and skill-gap among health care workers and business factors at the facility level. Training, support and regulatory interventions are required in order to improve the antimicrobial dispensing practices in community drug outlets, which will then lead to rational use of antimicrobials in the community and finally limit the rise of antimicrobial resistance.

## Data Availability

The datasets generated and/or analyzed during this study are available from the corresponding author upon reasonable request.

## Abbreviations

ADDOs: Accredited Drug Dispensing Outlets
ASPs: Antibiotics Stewardship Programs
AMR: Antimicrobial Resistance
KAP: Knowledge, Attitude and Practice
RRHs: Regional Referral Hospitals
NAPAR: National Action Plan on Antimicrobial Resistance
